# Identification of HLA-DRPheβ47 as the susceptibility marker of hypersensitivity to beryllium in individuals lacking the berylliosis-associated supratypic marker HLA-DPGluβ69

**DOI:** 10.1186/1465-9921-6-94

**Published:** 2005-08-14

**Authors:** Massimo Amicosante, Floriana Berretta, Milton Rossman, Richard H Butler, Paola Rogliani, Ella van den Berg-Loonen, Cesare Saltini

**Affiliations:** 1 Department of Internal Medicine, University of Rome "Tor Vergata", Rome, Italy; 2 Pulmonary, Allergy and Critical Care Division, Department of Medicine, Hospital of the University of Pennsylvania, Philadelphia, Pennsylvania, USA; 3 Institute of Cell Biology, National Research Council, Monterotondo (Rome), Italy; 4 Tissue Typing Laboratory, University Hospital Maastricht, Maastricht, The Netherlands

## Abstract

**Background:**

Susceptibility to beryllium (Be)-hypersensitivity (BH) has been associated with HLA-DP alleles carrying a glutamate at position 69 of the HLA-DP β-chain (HLA-DPGlu69) and with several HLA-DP, -DQ and -DR alleles and polymorphisms. However, no genetic associations have been found between BH affected subjects not carrying the HLA-DPGlu69 susceptibility marker.

**Methods:**

In this report, we re-evaluated an already described patient populations after 7 years of follow-up including new 29 identified BH subjects. An overall population 36 berylliosis patients and 38 Be-sensitization without lung granulomas and 86 Be-exposed controls was analysed to assess the role of the individual HLA-class II polymorphisms associated with BH-susceptibility in HLA-DPGlu69 negative subjects by univariate and multivariate analysis.

**Results:**

As previously observed in this population the HLA-DPGlu69 markers was present in higher frequency in berylliosis patients (31 out of 36, 86%) than in Be-sensitized (21 out of 38, 55%, p = 0.008 vs berylliosis) and 41 out of 86 (48%, p < 0.0001 vs berylliosis, p = 0.55 vs Be-sensitized) Be-exposed controls.

However, 22 subjects presenting BH did not carry the HLA-DPGlu69 marker. We thus evaluated the contribution of all the HLA-DR, -DP and -DQ polymorphisms in determining BH susceptibility in this subgroup of HLA-Glu69 subjects. In HLA-DPGlu69-negatives a significant association with BH was found for the HLA-DQLeu26, for the HLA-DRB1 locus residues Ser13, Tyr26, His32, Asn37, Phe47 and Arg74 and for the HLA-DRB3 locus clusterized residues Arg11, Tyr26, Asp28, Leu38, Ser60 and Arg74. HLA-DRPhe47 (OR 2.956, p < 0.05) resulting independently associated with BH. Further, Be-stimulated T-cell proliferation in the HLA-DPGlu69-negative subjects (all carrying HLA-DRPhe47) was inhibited by the anti-HLA-DR antibody (range 70–92% inhibition) significantly more than by the anti-HLA-DP antibody (range: 6–29%; p < 0.02 compared to anti-HLA-DR) while it was not affected by the anti-HLA-DQ antibody.

**Conclusion:**

We conclude that HLA-DPGlu69 is the primary marker of Be-hypersensitivity and HLA-DRPhe47 is associated with BH in Glu69-negative subjects, likely playing a role in Be-presentation and sensitization.

## Background

Due to its unique chemical-physical properties, beryllium (Be) compounds continue to be used in aerospace, ceramics, defence, electronics and telecommunication industries where inhalation of Be dust is the cause of Be-hypersensitivity (BH) in susceptible individuals [1]. Among subjects developing Be-hypersensitivity, all show sensitization, i.e. T-cell reactivity to Be revealed by either a blood or a bronchalveolar lavage cell test. Less than 50% of subjects with BH present which chronic disease [1-3] i.e., with chronic granuloma formation in the lung maintained by the accumulation in the lower respiratory tract of CD4+ T-cells responding to Be as a specific antigen/hapten [4], presenting an effector-memory phenotype [5,6] and producing Th1 cytokines upon Be stimulation [4-6].

The observation that beryllium disease affects only 1 to 16% of Be-exposed individuals led to the hypothesis that genetic susceptibility may play an important role in the pathogenesis of this disease [1]. In 1993, the HLA-DP supratypic variant characterized by a glutamic acid at position 69 of the HLA-DP molecule β chain (DPGlu69) was identified as a genetic marker of susceptibility to BH, an observation subsequently confirmed by seven independent studies [7-14]. Two independent studies have also identified the HLA-DPGlu69 marker as the immune response gene responsible for presentation of Be to Be-specific T-cells [15,16] and an immunochemical study has suggested that the structural basis for Be presentation by the HLA-DPGlu69 positive molecule is in its unique ability to bind beryllium with high affinity possibly in the context of a coordination bond formed by the contribution of other electron donor groups present in the fourth pocket of the peptide binding groove of the HLA-DP molecule [17]. Further, antibody inhibition studies have shown that Be-presentation to blood and lung T-cells in DPGlu69-positive subjects is inhibited almost exclusively by anti-HLA-DP antibodies [16,18], strongly indicating HLA-DPGlu69 as the immune response gene used by DPGlu69-positive subjects i.e., about 80% of the BH affected population [7-14].

In contrast, the HLA gene which might function as the immune response gene in DPGlu69-negative BH-affected subjects i.e., in the remaining 20% of the BH affected population, has not yet been determined.

Previous studies have identified the HLA-DRB1 alleles belonging to the *01 group [13] as negatively associated with berylliosis, while the HLA-DRB1 variants Ser11 [12], Tyr26 [10], Asn37 [12], Glu71 [12] and Arg74 [10] and the HLA-DQ variant Gly86 [12] were positively associated with BH. Analysis of the role of these markers has, however, been hampered by the small size of the populations examined in most studies. In all studies published so far, the putative susceptibility markers covered only 40 to 50% of the DPGlu69-negative subjects. In this context, our previous study [10] on 45 individuals affected by beryllium sensitization with or without demonstrable lung granulomas, showed that HLA-DR Arg74 and Tyr26 were associated with sensitization without lung granulomas, and HLA-DP Glu69 with sensitization accompanied by lung granulomas, thereby suggesting a different role for Glu69 and these markers [10]. However, in the HLA-DPGlu69 negative subjects reported in the Saltini et al. study population, HLA-DR Arg74 and Tyr26 were expressed only by 11 out of 19 DPGlu69 negative sensitized subjects 10 of which without and one with demonstrable lung granulomas [10]. In another study in the field conducted by Rossman et al. [12] evaluating 56 BH affected subjects, four out of seven DPGlu69 negative patients carried either DRAsn37, DRGlu71 or DQGly86 [12]. Finally, Maier and co-workers [13], in 19 HLA-DP Glu69-negative BH subjects, found that HLA-DRB1*13 alleles were associated with BH susceptibility; however, they were only expressed by 12 of these subjects.

In order to search for this(these) disease associated immune response gene(s), we re-evaluated a previously described population [10] after a follow up of 7 years that allowed us to extend the study to other 29 newly identified BH subjects (14 with biopsy proven lung granulomas and 15 with Be-sensitization without lung granulomas) for a total number of 74 BH subjects and 86 Be-exposed controls. This panel included a sufficiently large number of DPGlu69-negative subjects to analyze phenotypic frequencies of all aminoacid variants of the HLA-DPB1, -DQB1, -DRB1, -DRB3, -DRB4 and DRB5 genes in BH affected and Be exposed controls.

## Methods

### Study population

The study population, already described in part in a previous study [10], includes 86 Be exposed healthy controls and 74 subjects affected by beryllium hypersensitivity, all working in the same beryllium manufacturing plant, 45 of whom (23 Be-sensitized subjects and 22 berylliosis affected i.e., Be-sensitized subjects with biopsy proven lung granulomas) have been already described in the Saltini et al. report [10]. Study subjects are categorized as (i) Be-exposed controls, when having negative blood Be-LPT test, (ii) Be-sensitized, when having 2 blood BeLPT positive tests and (iii) berylliosis-affected when having 2 blood BeLPT positive tests and/or biopsy proven lung granulomas [10]. While 4 control subjects were diagnosed with beryllium sensitization and 3 with berylliosis during the 7-years follow up, none of the subjects in the previous study progressed from sensitization to berylliosis.

Overall the population in this report included 36 berylliosis (age 40 ± 7 years; 33 Caucasians, 2 African-Americans and 1 Asian; 32 males and 4 females; mean duration of Be-exposure 11 ± 7 years) and 38 showed Be-sensitization without lung granulomas detected by trans-bronchial biopsy (age 43 ± 9 years; 37 Caucasians and 1 Afro-American; 31 males and 7 females; mean age of Be-exposure 17 ± 9 years) and 86 Be-exposed controls (age 44 ± 9 years; 81 Caucasians, 2 African-American, 2 Hispanics, 1 Asian; 71 males and 15 females; mean duration of Be-exposure 16 ± 11 years).

### High resolution HLA class II typing

High resolution HLA-class II typing for the HLA-DPB1, DQB1, DRB1, DRB3, DRB4, DRB5 loci were performed by standard protocols as already reported [10].

### Beryllium lymphocyte proliferation test (Be-LPT)

The Be-LPT as measures of the T-cell lymphocytes response against Be in peripheral blood has been performed by standard method [19]. Briefly, peripheral blood mononuclear cells (PBMC) were tested against three doses beryllium sulfate (BeSO_4_*4H_2_O) at 1, 10, and 100 μM at 3 and 5 days. T-cell proliferation were evaluated by tritium (^3^H) labelled thymidine incorporation and a stimulation index (SI) calculated as the ratio of the radioactivity (counts per minute) of beryllium-exposed cell cultures to the count rate of unstimulated cultures. A test was defined as abnormal when two or more stimulation index values of six possible values exceeded the normal standard ratio of 3.0 (stimulated to unstimulated) as the cut-off.

### Lymphocyte proliferation to Be salt and monoclonal antibody (MoAb) inhibition of lymphocyte activation

T-cell proliferation in response to BeSO_4 _and inhibition by anti-HLA class II MoAbs were performed as previously described [18]. Briefly, PBMCs obtained from patients with BH were isolated from heparinized whole blood by density centrifugation on Ficoll Hypaque gradient. PBMCs (2 × 10^5 ^cells/well) were then cultured in 96-well flat-bottomed microtiter plates in RPMI 1640 tissue culture medium supplemented with 2 mM L-glutamine, 10% fetal bovine serum, 100 U/ml penicillin, and 100 μg/ml streptomycin in the presence of beryllium sulfate (BeSO_4_*4H_2_O) at 10–50–100 μM (all reagents form Sigma Co., St. Louise, MO). Phytohemoagglutinin (PHA, 5 μg/ml, Sigma) and *Candida albicans *(10 μg/ml) were used as positive controls. T lymphocyte proliferation was measured by [3H]TdR incorporation. Cells were pulsed with 1 μCi of [^3^H]TdR (Amersham International, Amersham, UK) after 5 days of culture and harvested onto glass fiber filters 18 hours later. Proliferation was measured as ^3^H-TdR incorporation by liquid scintillation spectroscopy and the test was scored as positive in the presence of a greater than twofold proliferation index. Protein-A sepharose purified MoAb directed against HLA-DR (L243) [15], HLA-DP (B7/21) [15], HLA-DQ (L2) [15], HLA-class I (W6/32) [15] were used at increasing concentrations (10, 20 and 50 μg/ml) to inhibit antigen presentation and lymphocyte proliferation as previously described [4,15,18]. The 19 kDa *Mycobacterium tuberculosis *(MTB19) protein monoclonal antibody HYT6 [20] was used as control.

### Statistical analysis

Statistical analysis was carried out as previously described [10,21,22]. Phenotypic frequency data are expressed as percentages with Odds Ratio (OR) with respect to the Be-exposed control group when appropriate. Comparisons between phenotypic frequencies in the study groups were done by χ^2 ^test with the Yates correction where necessary. Linkage disequilibrium analysis was carried out as previously described [20]. Forward and stepwise multiple logistic regression multivariate analysis were applied for identifying independent parameter(s) in multiple comparisons. Be-stimulated lymphocyte proliferation data are expressed as mean ± standard deviation of the mean (SD). Comparisons between groups in Be-lymphocyte proliferation data were done using the Student's t test with Welch's correction when appropriate. All the statistical analysis were carried out with the SPSS (SPSS inc., Chicago, IL) and GraphPad Prism (GraphPad Software Inc., San Diego, CA) packages.

## Results

The allelic frequencies for HLA-DPB1, DQB1 and DRB1, 3, 4 and 5 in general population are reported in the tables 1–4 of the additional file (supported material.pdf).

Similarly to the previous study on this population [10] the HLA-DPGlu69 marker was carried with higher frequency by berylliosis affected (31 out of 36, 86%) than subjects with Be-sensitization without granuloma (21 out of 38, 55%, p = 0.008 vs berylliosis affected) and Be-exposed controls (41 out of 86, 48%, p < 0.0001 vs berylliosis affected, p = 0.55 vs Be-sensitized).

The HLA-DPGlu69 has been previously proposed as a marker of progression from the sensitization state to the lung granulomatous reaction of chronic beryllium disease [10,13]. In this study population there were no cases of progression from systemic sensitization to lung disease notwithstanding the substantial follow-up period of 7.0 ± 3.7 years from the first positive Be-LPT test, while 4 control subjects were diagnosed with beryllium sensitization and 3 with berylliosis during the 7-years follow up. Hence, we could not directly look at the HLA-DPGlu69 association with disease progression. In addition, the frequency of the HLA-DPGlu69 homozygosity, another marker associated with disease progression [13] was higher in the disease affected population compared to the sensitized and the Be-exposed control population [5 out of 86 healthy exposed controls (5.8%), 3 out of 38 sensitized without disease (7.9%; p = 0.97 compared to controls) and 8 out of 36 disease affected (22.2%; p = 0.06 compared to controls; p = 0.10 compared to the sensitized)] although the difference was not statistically significant.

A total number of 22 BH subjects (17 Be sensitized and 5 berylliosis affected) and 45 Be-exposed controls were HLA-DPGlu69 negative. They did not differ from the DPGlu69-positive (neither the BH affected nor the Be-exposed controls) in terms of gender, ethnicity, age or length of Be-exposure (p > 0.05, all comparisons). The allelic frequencies for HLA-DPB1, DQB1 and DRB1, 3, 4 and 5 in HLA-DPGlu69 negative subjects are reported in the tables 5–8 of the additional file (supported material.pdf).

This subgroup of HLA-DPGlu69 negative subjects was analyzed for the distribution of all HLA class II polymorphic aminoacid residues, with the exception of HLA-DPGlu69, by univariate analysis. No associations were found between any of the HLA-DP polymorphic residues and BH. Strikingly, among the polymorphic residues of the HLA-DR β-chain coded for by the HLA-DRB1 locus, residues Ser13, Tyr26, His32, Asn37, Phe47 and Arg74 were associated with BH (Table 1). Similarly, the HLA-DR β-chain HLA-DRB3 locus polymorphic residues Arg11, Tyr26, Asp28, Leu38, Ser60 and Arg74 were found associated to BH (Table 1). No polymorphisms associated with BH were found in the HLA-DRB4 and DRB5 loci. Finally, a statistically significant association with BH in HLA-DPGlu69 negatives was found for the HLA-DQB1 gene polymorphic residue Leu26 (Table 1).

**Table 1:** Phenotypic frequencies of the polymorphisms found associated with Be-hypersensitivity in HLA-DPGlu69 negative subjects.

	**Be-exposed controls (n = 45)**	**Be-Hypersensitive (n = 22)**		
**HLA-DRB1 polymorphisms**^1^	**N positive subjects (%)**	**N positive subjects (%)**	**OR**^2^	**p**^3^

Ser13	28 (62.2%)	20 (90.9)	6.07	0.015
Tyr26	7 (15.6%)	10 (45.5%)	4.52	0.009
His32	21 (46.7%)	17 (77.3%)	3.89	0.017
Asn37	17 (37.8%)	16 (72.7%)	4.39	0.007
Phe47	30 (66.7%)	21 (95.5%)	10.50	0.011
Arg74	7 (15.6%)	10 (45.5%)	4.52	0.009
				
**HLA-DQB1 polymorphism**^4^				
Leu26	32 (71.1%)	21 (95.5%)	8.53	0.021
				
**HLA-DRB3 polymorphisms**^5^	**N = 30**	**N = 17**		

Arg11	8 (26.7%)	12 (70.6%)	6.60	0.008
Tyr26	8 (26.7%)	12 (70.6%)	6.60	0.008
Asp28	8 (26.7%)	12 (70.6%)	6.60	0.008
Leu38	8 (26.7%)	12 (70.6%)	6.60	0.008
Ser60	8 (26.7%)	12 (70.6%)	6.60	0.008
Arg74	8 (26.7%)	12 (70.6%)	6.60	0.008

1. HLA-DRB1 polymorphisms found associated with Be-hypersensitivity in HLA-DPGlu69 negative subjects among the overall HLA-DRB1 polymorphic variants analyzed at positions: 9, 10, 11, 12, 13, 16, 26, 28, 30, 32, 37, 38, 47, 57, 58, 60, 67, 70, 71, 73, 74, 77, 85, 86.

2. Odds ratio with respect to Be-exposed controls.

3. p value (χ^2 ^analysis) with respect to Be-exposed controls.

4. HLA-DQB1 polymorphisms found associated with Be-hypersensitivity in HLA-DPGlu69 negative subjects among the overall HLA-DQB1 polymorphic variants analyzed at positions: 9, 13, 14, 23, 26, 28, 30, 37, 38, 45, 46, 47, 52, 53, 55, 56, 57, 66, 67, 70, 71, 74, 75, 77, 84, 85, 86, 87, 89, 90.

5. HLA-DRB3 polymorphisms found associated with Be-hypersensitivity in HLA-DPGlu69 negative subjects among the overall HLA-DRB3 polymorphic variants analyzed at positions: 8, 11, 26, 28, 30, 37, 38, 39, 51, 57, 58, 60, 67, 74, 77, 86. No polymorphisms were found associated to BH in HLA-DPGlu69 negatives in the HLA-DRB4 and DRB5 loci and HLA-DP locus.

However, as a linkage disequilibrium could exist between the HLA-DRB1 gene coded residues and other HLA-DRB1, -DRB3 and -DQB1 loci, in order to identify the independently associated residue(s) in the HLA-DPGlu69 negative BH subjects, multiple logistic regression models were carried out on all the HLA variants above. As a result, only HLA-DRPhe47 (OR 2.956, p < 0.05) was identified as independently associated with BH in the HLA-DPGlu69-negative subgroup, hence suggesting that the other HLA-DRB1, -DRB3 and -DQB1 loci shown in table 1 could be associated with BH due to linkage disequilibrium with HLA-DRPhe47. Of the 22 HLA-DPGlu69 negative subjects with BH, 21 were HLA-DRPhe47 (16 Be sensitized and 5 berylliosis affected) and only one, a Be sensitized individual, was HLA-DPGlu69 and HLA-DRPhe47 negative.

Further, in order to identify which of the HLA isotypic molecules associated with BH could function as the restriction elements of Be-stimulated T-cell proliferation in HLA-DPGlu69-negative subjects, we analyzed PBMC proliferation in response to BeSO_4 _in a subgroup of 15 BH affected subjects, using antibodies directed against HLA-DR, HLA-DQ and HLA-DP as probes to identify the antigen presentation restricting molecule.

**Figure 1:**
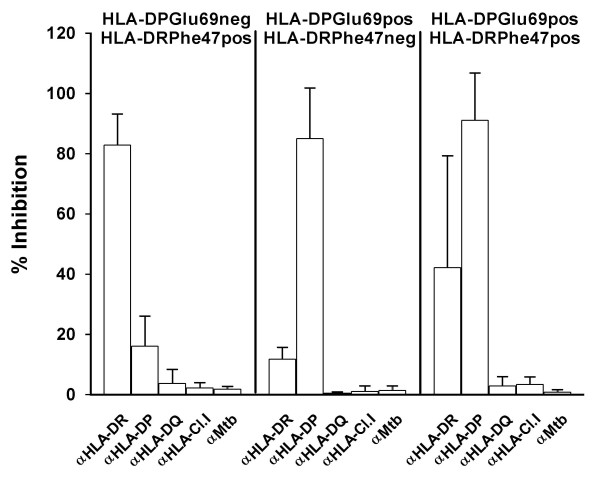
Inhibition of beryllium (BeSO_4_)-induced proliferation, by MoAbs directed against HLA-DR, HLA-DP, HLA-DQ, HLA-class I and the 19 kDa *M. tuberculosis *protein in PBMC from BH subjects carrying or not the HLA-DPGlu69 and the HLA-DRPhe47 markers. They were 4 HLA-DPGlu69 negative/HLA-DRPhe47 positive (3 sensitized and 1 berylliosis affected), 3 HLA-DPGlu69 positive/HLA-DRPhe47 negative (0 sensitized and 3 berylliosis affected) and 8 HLA-DPGlu69 positive/HLA-DRPhe47 positive (3 sensitized and 5 berylliosis affected). On the ordinate is shown the percentage of inhibition (with respect to the MoAbs untreated cells) of T-cell proliferation obtained by co-culturing the PBMC from berylliosis patients with BeSO_4 _in the presence of each MoAb reported on the abscissa (anti-HLA-DR: DR, anti-HLA-DP: DP, anti-HLA-DQ: DQ, anti-HLA-class I: C.I, anti-19 kDa *M. tuberculosis: *Mtb).

In four HLA-DPGlu69-negatives, Be-stimulated T-cell proliferation was inhibited by the anti-HLA-DR MoAb (range 70–92% inhibition) significantly more than by the anti-HLA-DP MoAb (range: 6–29%; p < 0.02 compared to anti-HLA-DR) while it was not affected at all by the anti-HLA-DQ, anti-HLA class I or the anti-MTB19 control antibody (Figure 1), suggesting a role for the HLA-DR molecule in the presentation of Be in the HLA-DPGlu69-negative subjects. All the four HLA-DPGlu69-negative subjects carried the HLA-DRPhe47 polymorphism.

In contrast, in the three subjects who were HLA-DPGlu69 positive and HLA-DRPhe47 negative, proliferation was completely inhibited by the anti-HLA-DP MoAb (range 68–100%) but not by anti-HLA-DR (range 7–14%, p < 0.05 compared to anti-HLA-DP), nor by anti-HLA-DQ, anti-HLA class I or the anti-MTB19 control antibody (Figure 1). Finally, in the eight subjects carrying both HLA-DPGlu69 and HLA-DRPhe47, the proliferative response to BeSO_4 _was always inhibited by the anti-HLA-DP (range: 63–100%) and variable inhibited by anti-HLA-DR antibodies (range: 0–94%), with the inhibition by anti HLA-DP being significantly stronger than the anti HLA-DR (paired t-test, p < 0.01).

## Discussion

Similarly to our previous report [10] and consistently with the more recent study by McCanlies et al. [14], the re-evaluation of this patient population shows a higher prevalence of HLA-DPGlu69 among the subjects with lung granulomas compared to the Be-sensitized without lung involvement (86% vs 55%, p = 0.008). However, not having identified any case of disease progression from beryllium sensitization to lung disease during the 7.0 years follow-up, we could not formally assess the association of the HLA-DPGlu69 marker with progression from sensitization to disease, although the knowledge that HLA-DPGlu69 is the primary immune response gene of beryllium hypersensitivity [15-17] makes it attractive to hypothesize that the gene might induce a stronger immune reaction hence inducing granuloma formation as suggested by Maier et al. [13] in which 11 out of 12 subjects progressed from sensitization to disease status were HLA-DPGlu69 positives [13], as well as in more recent publication in which Be-sensitized subjects progress to berylliosis at a rate of 6–8% per year [23]. However, it could be also considered that all these data could be inferred by the possibility of misdiagnosis in the identification of the Beryllium induced granuloma.

The finding that a sizeable fraction of BH affected subjects, varying from 3 to 27% in published reports [7-14], do not carry HLA-DPGlu69 has indicated that other HLA molecules may provide the restriction element of Be-stimulated T-cell proliferation and may be implicated in the pathogenesis of susceptibility to BH.

In this regard, with all the limitations imposed by the need of performing specific antigen presentation studies using specific reagents such as HLA-DRPhe47 restricted antigen presenting cells and/or HLA-DRPhe47-engineered transfectants as already made for HLA-DPGlu69 [15], the T-cell studies using isotype-specific inhibition of BeSO_4_-stimulated T-cell proliferation with anti-HLA isotype specific antibodies in HLA typed subjects support the notion that HLA-DR genes are implicated in beryllium presentation in HLA-DPGlu69-negative. A role for HLA-DRPhe47 in Be presentation is conceivable, and the data presented suggest it.

In fact, multiple analysis has indicated that the HLA-DR aminoacid variants Ser13, Tyr26, His32, Asn37, Arg74 are indirectly associated with beryllium hypersensitivity due to linkage disequilibrium with Phe47. It is worth noticing that this analysis accounts for the association of HLA-DRArg74 and HLA-DRTyr26 with beryllium sensitization found in a previous study [10]. These markers were identified for their positive and negative association of HLA-DR alleles with disease or sensitization using univariate analysis in the overall population analyzed [10], while in this re-evaluation of the same study population after 7-years of follow up including more subjects with Be-sensitization and disease we could use multiple logistic regression multivariate analysis on HLA-DPGlu69-negative subjects only. The fact that the alleles of the HLA-DRB1*03 group (alleles found associated with Be-sensitization and not disease in the previous evaluation of this study population [10]) carrying almost exclusively the HLA-DRArg74 and HLA-DRTyr26 are also carrying HLA-DRPhe47 support the notion of the linkage disequilibrium between them. Further, only a fraction of HLA-DPGlu69-negative carry only HLA-DRArg74 and -DRTyr26 (10 out of 22; 45.5%) while all except one (21 out of 22, 95.5%) carry HLA-DRPhe47 suggesting that more than an allele or set of them is a residue and the role play in the HLA-class II peptide binding pocket where he is mapping involved in the Be-presentation to T-cell determining Be-susceptibility.

Consistent with the T-cell antibody inhibition study, multiple regression analysis also indicates that the association between the HLA-DQ marker Leu26 and BH is attributable to linkage disequilibrium between HLA-DR and HLA-DQ loci [24]. It is well known that the HLA-DQLeu26 residue is expressed by all the HLA-DQB1*02 alleles and most of *03 and *06 alleles that are in linkage with the HLA-DRB1*03, 11, 12, 13 or 15 alleles which, in turn, express HLA-DRPhe47. Similar to our data, Maier and coworkers [13] obtained evidence for an association of HLA-DQB1*06, a Leu26 expressing group of alleles, with BH in HLA-DPGlu69-negative subjects. They too attributed the increased frequency of this HLA-DQ marker to linkage disequilibrium with HLA-DR and in particular to HLA-DR*13 alleles, a group of alleles expressing Phe47 [13].

The data of this study take the above observations [10,13] a step further by suggesting that the HLA-DR gene, possibly the HLA-DRPhe47 supratypic variant, ought to play a functional role in Be presentation, as this (Tyr/Phe 47) polymorphism is known to be important for peptide binding and presentation to T-cells [25,26]. Interestingly, HLA-DRPhe47 has been also implicated in susceptibility to the histopathological alike of berylliosis, sarcoidosis, in a very large case control population study [21].

**Figure 2:**
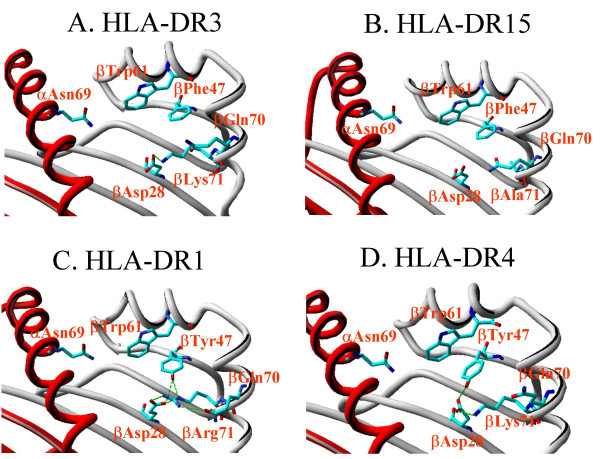
Analysis of the H-bond network in the pocket 7, the peptide binding pocket where the HLA-DR residue β47 is mapping, of HLA-DR molecules carrying HLA-DRPhe47 (Panel A: HLA-DR3 and Panel B: HLA-DR15) or its counterpart Tyr47 (Panel C: HLA-DR1 and Panel D: HLA-DR4). Molecular modelling of the PDB entry crystal structures (HLA-DR3: 1A6A; HLA-DR15: 1BX2; HLA-DR1: 1AQD; HLA-DR4: 2SEB) have been evaluated with the SwissPDB viewer v3.7b2 software (free available at ). The HLA-DR α-chain backbone is reported in red colored ribbon style, while the HLA-DR β-chain backbone is reported in grey colored ribbon. Aminoacids are colored in CPK style (C: light blue; O: red; N: blue) and residue names are reported in red. H-bonds were computed with the SwissPDB viewer (H-bond detection threshold: 1.20–2.76 A when Hydrogen is present and 2.19–3.30 A when Hydrogen is absent) and are shown as green dashed lines. All the aminoacids presenting electron donor groups in the pocket 7, of HLA-DR1, -DR3, -DR4 and -DR15, putatively capable of coordinating Be are shown (residues α69, β28, β61, β70 and β71). In the HLA-DR3 crystal structure (Panel A) with the presence of Phe47 only one of the two terminal oxygens of Aspβ28 is engaged in a H-bond network with Lys71, leaving four other contacts points for co-ordinating Be (specifically residues αAsn69, βAsp28, βTrp61, βGln70). A similar pattern is present in HLA-DR15 (Panel B) where, with the presence of Phe47, no H-bonds are present leaving 5 electron donor groups available for Be coordination (specifically one electron donor group for each residue αAsn69, βTrp61, βGln70 and two electron donor groups for βAsp28). When Tyr47, the HLA-DRPhe47 counterpart, is present in HLA-DR molecules as in HLA-DR1 (panel C) and HLA-DR4 (Panel D), the H-bond network of pocket 7 results dramatically modified. Specifically, Tyr47 engages in a H-bond network with residues Asp28 and Arg71 in HLA-DR1 (Panel C) or Asp28 and Lys71 in HLA-DR4 (Panel D). As a consequence there is reduced availability of electron donor groups capable to coordinate Be.

How might the HLA-DRPhe47 molecule bind beryllium? Potolicchio et al. have shown that cobalt binds directly to polymorphic residue(s), likely Glu69 and/or Glu56, of the HLA-DP molecule [27], and this model may apply to beryllium interaction with HLA-DP. On the other hand, Lu et al. have recently reported that nickel interacts both with the HLA-DR backbone, with the non-polymorphic His in position 81 of the HLA-DR β-chain, and with a bound peptide [28]. This latter mechanism could also be envisioned for beryllium interaction with HLA-DRPhe47. Residue 47 of the HLA-DR β-chain is located in pocket 7, together with residues 65 and 69 of the α-chain and residues 28, 30, 61, 67, 70 of the β-chain, involving to some extent residues 71 and 74 of the β-chain which however do primarily contribute to pocket 4 [29-32]. In pocket 7, besides residue 47, only residues 28, 67 and 70 of the β chain are polymorphic. Interestingly, all of the alleles carrying the HLA-DRPhe47 in the HLA-DPGlu69-negative BH subjects, expressed always an aspartic acid residue 28 of the β-chain and either an aspartic acid or a glutamine, both residues which, together with the asparagine and tryptophan present at the non polymorphic residues 69 of the α-chain and 61 of the β-chain, could coordinate the positive charge of Be for presentation to Be-specific T-cells. The spatial relationships between the residues potentially involved may be defined precisely from the crystal structure of HLA-DR3 and HLA-DR15 [30,32] (Figure 2, panels A-B). As seen from the crystal structure, when Phe47 in pocket 7 is substituted for by Tyr47 in the HLA-DR1 and HLA-DR4 molecules, the hydrogen of the Tyr47 hydroxyl is engaged in a hydrogen bound network with the aspartic acid at position 28 and glutamine at position 70 of the β-chain [31,33] (Figure 2, panels C-D), thereby preventing their participation in coordinating the charge of the Be ion. Thus, the presence of Phe47 in pocket 7 of the HLA-DR molecule could favor Be binding. Furthermore, the HLA-DR molecules carrying Phe47 could coordinate Be together with a bound peptide with a mechanism similar to that described for nickel [28]. A number of considerations are likely to support this hypothesis. First, the distance between the different electron donor groups in the crystal structure of HLA-DR molecules carrying Phe47 lies between 6.7 and 12.6 Å [30,32] i.e., very close to the upper limits of a coordination bond with Be. Second, the two electron donor (non-polymorphic) residues α69 (Asn) and β61 (Trp) of HLA-DR pocket 7 are known to be involved in the formation of H-bonds with the backbone of the peptide antigen [29] and would therefore be unable to directly coordinate Be. Finally, the pocket 7 of HLA-DR allelic variants carrying Phe47 are capable of binding, with higher affinity than pocket 7 carrying Tyr47, aminoacid side chains with electron donor groups such as Asn, His, Met, Trp and Tyr [34].

## Conclusion

In conclusion, both the HLA typing and the *in vitro *T-cell data in this study indicate a role for HLA-DR genes in determining susceptibility to beryllium hypersensitivity among individuals not expressing the HLA-DPGlu69 variant. The typing data point to the HLA-DRPhe47 supratypic variant as the susceptibility gene in this sub-population, and analysis of the molecule's structure suggests that HLA-DRPhe47 could bind beryllium and present it to T-cells, using a mechanism different from what used by HLA-DPGlu69. Together, HLA-DPGlu69 and HLA-DRPhe47 could account for susceptibility in almost 100% of the affected population.

## Competing interests

1. Massimo Amicosante: I declare that I have NOT financial and non-financial competing interests in relation to this manuscript.

2. Floriana Berretta: I declare that I have NOT financial and non-financial competing interests in relation to this manuscript.

3. Milton Rossman: I declare that I have NOT financial and non-financial competing interests in relation to this manuscript.

4. Richard H. Butler: I declare that I have NOT financial and non-financial competing interests in relation to this manuscript.

5. Paola Rogliani: I declare that I have NOT financial and non-financial competing interests in relation to this manuscript.

6. Ella van den Berg-Loonen: I declare that I have NOT financial and non-financial competing interests in relation to this manuscript.

7. Cesare Saltini: I declare that I have NOT financial and non-financial competing interests in relation to this manuscript.

## Authors' contributions

MA carried out the analysis of the HLA class II polymorphisms, participated at the functional studies of HLA class II restriction of response to beryllium, participated at the study design and drafted the manuscript. FB carried out the functional studies of HLA class II restriction of response to beryllium and participated the analysis of the HLA class II polymorphisms. MR participated in the design of the study, contributed to the data analysis review and control and contributed to draft the manuscript. RHB performed the HLA class II modeling evaluation and beryllium-binding hypothesis to HLA-DR molecules carrying Phe47. PR performed the study population re-evaluation. EvdBL performed the HLA class II high resolution typing. CS performed the study design, supervised the study population clinical follow up and analysis work and drafted the manuscript. All authors read and approved the final manuscript.

## Supplementary Material

Additional File 1 The additional file (supplemented material.pdf) includes 8 tables reporting the allelic frequency for HLA-DPB1, DQB1 and DRB1, 3, 4 and 5 both in general population (tables #1–4) and in the HLA-DPGlu69 negative subjects (tables #5–8). Click here for file
