# Supplementary material for: Identification of HLA-DRPheβ47 as the susceptibility marker of hypersensitivity to beryllium in individuals lacking the berylliosis-associated supratypic marker HLA-DPGluβ69
Source: Respir Res. 2005 Aug 14;6(1):94. doi: 10.1186/1465-9921-6-94 (PMC1198259; doi:10.1186/1465-9921-6-94)
Supplement: Additional File 1 — The additional file (supplemented material.pdf) includes 8 tables reporting the allelic frequency for HLA-DPB1, DQB1 and DRB1, 3, 4 and 5 both in general population (tables #1–4) and in the HLA-DPGlu69 negative subjects (tables #5–8). [file 1465-9921-6-94-S1.doc]

# SUPPLEMENTED MATERIAL TO MS 2917410996553155

**Identification of HLA-DRPheβ47 as the susceptibility marker of hypersensitivity to beryllium in individuals lacking the berylliosis-associated supratypic marker HLA-DPGluβ69.**

**Supplemented material table 1. Frequencies of the HLA-DPB1 alleles in the study populations**.

|  | **Be-exposed controls** | | **Be-hypersensitives**  **(CBD+Be-sensitized without disease)** | | **CBD** | | **Be-sensitized without disease** | |
| --- | --- | --- | --- | --- | --- | --- | --- | --- |
| **Allele** | **N Alleles**  **(172)** | **%** | **N Alleles**  **(148)** | **%** | **N Alleles (72)** | **%** | **N Alleles**  **(76)** | **%** |
| **0101** | 6 | 3.5% | 6 | 4.1% | 2 | 2.8% | 4.00 | 5.3% |
| **0201** | 36 | 20.9% | 40 | 27.0% | 25* | 34.7% | 15.00 | 19.7% |
| **0202** | 1 | 0.6% | 0 | 0.0% | 0 | 0.0% | 0 | 0.0% |
| **0301** | 15 | 8.7% | 8 | 5.4% | 4 | 5.6% | 4.00 | 5.3% |
| **0401** | 65 | 37.8% | 41 | 27.7% | 13* | 18.1% | 28.00 | 36.8% |
| **0402** | 26 | 15.1% | 13 | 8.8% | 8 | 11.1% | 5.00 | 6.6% |
| **0501** | 3 | 1.7% | 6 | 4.1% | 1 | 1.4% | 5.00 | 6.6% |
| **0601** | 1 | 0.6% | 4 | 2.7% | 1 | 1.4% | 3.00 | 3.9% |
| **0901** | 0 | 0.0% | 2 | 1.4% | 2 | 2.8% | 0.00 | 0.0% |
| **1001** | 5 | 2.9% | 6 | 4.1% | 4 | 5.6% | 2.00 | 2.6% |
| **1101** | 3 | 1.7% | 3 | 2.0% | 1 | 1.4% | 2.00 | 2.6% |
| **1301** | 1 | 0.6% | 4 | 2.7% | 2 | 2.8% | 2.00 | 2.6% |
| **1401** | 2 | 1.2% | 3 | 2.0% | 3 | 4.2% | 0.00 | 0.0% |
| **1501** | 2 | 1.2% | 1 | 0.7% | 0 | 0.0% | 1.00 | 1.3% |
| **1601** | 1 | 0.6% | 3 | 2.0% | 1 | 1.4% | 2.00 | 2.6% |
| **1701** | 0 | 0.0% | 3 | 2.0% | 3 | 4.2% | 0.00 | 0.0% |
| **1901** | 1 | 0.6% | 2 | 1.4% | 2 | 2.8% | 0.00 | 0.0% |
| **2001** | 0 | 0.0% | 2 | 1.4% | 0 | 0.0% | 2.00 | 2.6% |
| **2301** | 1 | 0.6% | 1 | 0.7% | 0 | 0.0% | 1.00 | 1.3% |
| **3501** | 1 | 0.6% | 0 | 0.0% | 0 | 0.0% | 0.00 | 0.0% |
| **7701** | 1 | 0.6% | 0 | 0.0% | 0 | 0.0% | 0 | 0.0% |
| **7801** | 1 | 0.6% | 0 | 0.0% | 0 | 0.0% | 0 | 0.0% |

Notes

*: Uncorrected p<0.03 and Bonferroni’s corrected p>0.05 vs Be-exposed controls.

**Supplemented material table 2. Frequencies of the HLA-DQB1 alleles in the study populations.**

|  | **Be-exposed controls** | | **Be-hypersensitives**  **(CBD+Be-sensitized without disease)** | | **CBD** | | **Be-sensitized without disease** | |
| --- | --- | --- | --- | --- | --- | --- | --- | --- |
| **Allele** | **N Alleles**  **(172)** | **%** | **N Alleles**  **(148)** | **%** | **N Alleles**  **(72)** | **%** | **N Alleles**  **(76)** | **%** |
| **0201** | 24 | 14.0% | 33 | 22.3% | 15 | 20.8% | 18 | 23.7% |
| **0202** | 0 | 0.0% | 2 | 1.4% | 0 | 0.0% | 2 | 2.6% |
| **0301** | 37 | 21.5% | 23 | 15.5% | 11 | 15.3% | 12 | 15.8% |
| **0302** | 19 | 11.0% | 10 | 6.8% | 4 | 5.6% | 6 | 7.9% |
| **0303** | 8 | 4.7% | 8 | 5.4% | 4 | 5.6% | 4 | 5.3% |
| **0304** | 1 | 0.6% | 0 | 0.0% | 0 | 0.0% | 0 | 0.0% |
| **0402** | 4 | 2.3% | 2 | 1.4% | 0 | 0.0% | 2 | 2.6% |
| **0501** | 14 | 8.1% | 17 | 11.5% | 7 | 9.7% | 10 | 13.2% |
| **0502** | 4 | 2.3% | 1 | 0.7% | 0 | 0.0% | 1 | 1.3% |
| **0503** | 5 | 2.9% | 3 | 2.0% | 1 | 1.4% | 2 | 2.6% |
| **0601** | 2 | 1.2% | 0 | 0.0% | 0 | 0.0% | 0 | 0.0% |
| **0602** | 28 | 16.3% | 26 | 17.6% | 13 | 18.1% | 13 | 17.1% |
| **0603** | 14 | 8.1% | 15 | 10.1% | 12 | 16.7% | 3 | 3.9% |
| **0604** | 9 | 5.2% | 6 | 4.1% | 4 | 5.6% | 2 | 2.6% |
| **0609** | 3 | 1.7% | 2 | 1.4% | 1 | 1.4% | 1 | 1.3% |

**Supplemented material table 3. Frequencies of the HLA-DRB1 alleles in the study populations.**

|  | **Be-exposed controls** | | **Be-hypersensitives**  **(CBD+Be-sensitized without disease)** | | **CBD** | | **Be-sensitized without disease** | |
| --- | --- | --- | --- | --- | --- | --- | --- | --- |
| **Allele** | **N Alleles**  **(172)** | **%** | **N Alleles**  **(148)** | **%** | **N Alleles**  **(72)** | **%** | **N Alleles**  **(76)** | **%** |
| **0101** | 10 | 5.8% | 15 | 10.1% | 6 | 8.3% | 9 | 11.8% |
| **0102** | 2 | 1.2% | 1 | 0.7% | 0 | 0.0% | 1 | 1.3% |
| **0103** | 1 | 0.6% | 0 | 0.0% | 0 | 0.0% | 0 | 0.0% |
| **0301** | 13 | 7.6% | 24* | 16.2% | 9 | 12.5% | 15* | 19.7% |
| **0401** | 18 | 10.5% | 5* | 3.4% | 3 | 4.2% | 2 | 2.6% |
| **0402** | 1 | 0.6% | 2 | 1.4% | 0 | 0.0% | 2 | 2.6% |
| **0404** | 4 | 2.3% | 4 | 2.7% | 1 | 1.4% | 3 | 3.9% |
| **0405** | 3 | 1.7% | 1 | 0.7% | 0 | 0.0% | 1 | 1.3% |
| **0407** | 1 | 0.6% | 0 | 0.0% | 0 | 0.0% | 0 | 0.0% |
| **0408** | 2 | 1.2% | 0 | 0.0% | 0 | 0.0% | 0 | 0.0% |
| **0410** | 1 | 0.6% | 0 | 0.0% | 0 | 0.0% | 0 | 0.0% |
| **0701** | 17 | 9.9% | 18 | 12.2% | 10 | 13.9% | 8 | 10.5% |
| **0801** | 3 | 1.7% | 2 | 1.4% | 0 | 0.0% | 2 | 2.6% |
| **0806** | 0 | 0.0% | 2 | 1.4% | 2 | 2.8% | 0 | 0.0% |
| **0810** | 0 | 0.0% | 1 | 0.7% | 0 | 0.0% | 1 | 1.3% |
| **0901** | 0 | 0.0% | 1 | 0.7% | 0 | 0.0% | 1 | 1.3% |
| **1001** | 1 | 0.6% | 1 | 0.7% | 1 | 1.4% | 0 | 0.0% |
| **1101** | 14 | 8.1% | 9 | 6.1% | 5 | 6.9% | 4 | 5.3% |
| **1102** | 2 | 1.2% | 2 | 1.4% | 1 | 1.4% | 1 | 1.3% |
| **1103** | 5 | 2.9% | 1 | 0.7% | 1 | 1.4% | 0 | 0.0% |
| **1104** | 3 | 1.7% | 4 | 2.7% | 3 | 4.2% | 1 | 1.3% |
| **1201** | 1 | 0.6% | 2 | 1.4% | 1 | 1.4% | 1 | 1.3% |
| **1301** | 15 | 8.7% | 18 | 12.2% | 14*# | 19.4% | 4 | 5.3% |
| **1302** | 12 | 7.0% | 8 | 5.4% | 5 | 6.9% | 3 | 3.9% |
| **1303** | 4 | 2.3% | 2 | 1.4% | 0 | 0.0% | 2 | 2.6% |
| **1401** | 5 | 2.9% | 3 | 2.0% | 1 | 1.4% | 2 | 2.6% |
| **1406** | 1 | 0.6% | 0 | 0.0% | 0 | 0.0% | 0 | 0.0% |
| **1501** | 26 | 15.1% | 21 | 14.2% | 9 | 12.5% | 12 | 15.8% |
| **1502** | 3 | 1.7% | 0 | 0.0% | 0 | 0.0% | 0 | 0.0% |
| **1601** | 4 | 2.3% | 1 | 0.7% | 0 | 0.0% | 1 | 1.3% |

Notes

*: Uncorrected p<0.03 and Bonferroni’s corrected p>0.05 vs Be-exposed controls.

#: Uncorrected p<0.02 and Bonferroni’s corrected p>0.05 vs Be-sensitized without disease.

**Supplemented material table 4. Frequencies of the HLA-DRB3, -DRB4 and -DRB5 alleles in the study populations.**

|  | **Be-exposed controls** | | **Be-hypersensitives**  **(CBD+Be-sensitized without disease)** | | **CBD** | | **Be-sensitized without disease** | |
| --- | --- | --- | --- | --- | --- | --- | --- | --- |
| **Allele** | **N DRB3 alleles=77** | **%** | **N DRB3 alleles=77** | **%** | **N DRB3 alleles=40** | **%** | **N DRB3 alleles=33** | **%** |
| 3*0101 | 21 | 27.3% | 29 | 37.7% | 9 | 22.5% | 20*# | 60.6% |
| **3*0201** | 2 | 2.6% | 0 | 0.0% | 0 | 0.0% | 0 | 0.0% |
| **3*0202** | 42 | 54.5% | 36 | 46.8% | 26 | 65.0% | 10*# | 30.3% |
| **3*0301** | 12 | 15.6% | 8 | 10.4% | 5 | 12.5% | 3 | 9.1% |
|  |  |  |  |  |  |  |  |  |
|  | **N DRB4 alleles=48** |  | **N DRB4 alleles=31** |  | **N DRB4 alleles=14** |  | **N DRB4 alleles=17** |  |
| **4*0101** | 10 | 20.8% | 7 | 22.6% | 3 | 21.4% | 4 | 23.5% |
| **4*0103** | 38 | 79.2% | 24 | 77.4% | 11 | 78.6% | 13 | 76.5% |
|  |  |  |  |  |  |  |  |  |
|  | **N DRB5 alleles=34** |  | **N DRB5 alleles=22** |  | **N DRB5 alleles=9** |  | **N DRB5 alleles=13** |  |
| **5* 0101** | 28 | 82.4% | 21 | 95.5% | 9 | 100.0% | 12 | 92.3% |
| **5* 0102** | 2 | 5.9% | 0 | 0.0% | 0 | 0.0% | 0 | 0.0% |
| **5* 0202** | 4 | 11.8% | 1 | 4.5% | 0 | 0.0% | 1 | 7.7% |

Notes

*: Uncorrected p<0.02 and Bonferroni’s corrected p>0.05 vs Be-exposed controls.

#: Uncorrected p<0.02 and Bonferroni’s corrected p>0.05 vs CBD.

**Supplemented material table 5. Frequencies of the HLA-DPB1 alleles in the HLA-DPGlu69-negative population.**

|  | **Be-exposed controls** | | **Be-hypersensitives**  **(CBD+Be-sensitized without disease)** | |
| --- | --- | --- | --- | --- |
| **Allele** | **N allele (90)** | **%** | **N allele (44)** | **%** |
| 0101 | 3 | 3.3% | 2 | 4.5% |
| **0301** | 14 | 15.6% | 5 | 11.4% |
| **0401** | 43 | 47.8% | 19 | 43.2% |
| **0402** | 18 | 20.0% | 7 | 15.9% |
| **0501** | 3 | 3.3% | 4 | 9.1% |
| **1101** | 2 | 2.2% | 2 | 4.5% |
| **1401** | 2 | 2.2% | 1 | 2.3% |
| **1501** | 1 | 1.1% | 1 | 2.3% |
| **2001** | 0 | 0.0% | 2 | 4.5% |
| **2301** | 1 | 1.1% | 1 | 2.3% |
| **3501** | 1 | 1.1% | 0 | 0.0% |
| **7701** | 1 | 1.1% | 0 | 0.0% |
| **7801** | 1 | 1.1% | 0 | 0.0% |

**Supplemented material table 6. Frequencies of the HLA-DQB1 alleles in the HLA-DPGlu69-negative population**

|  | **Be-exposed controls** | | **Be-hypersensitives**  **(CBD+Be-sensitized without disease)** | |
| --- | --- | --- | --- | --- |
| Alleles | **N allele (90)** | **%** | **N allele (44)** | **%** |
| 0201 | 13 | 14.4% | 14 | 31.8%* |
| **0202** | 0 | 0.0% | 1 | 2.3% |
| **0301** | 17 | 18.9% | 4 | 9.1% |
| **0302** | 10 | 11.1% | 1 | 2.3% |
| **0303** | 5 | 5.6% | 2 | 4.5% |
| **0402** | 1 | 1.1% | 1 | 2.3% |
| **0501** | 9 | 10.0% | 4 | 9.1% |
| **0502** | 3 | 3.3% | 0 | 0.0% |
| **0503** | 4 | 4.4% | 2 | 4.5% |
| **0602** | 16 | 17.8% | 9 | 20.5% |
| **0603** | 6 | 6.7% | 3 | 6.8% |
| **0604** | 6 | 6.7% | 3 | 6.8% |

*: Uncorrected p<0.05 and Bonferroni’s corrected p>0.05 vs Be-exposed controls.

**Supplemented material table 7. Frequencies of the HLA-DRB1 alleles in the HLA-DPGlu69-negative population.**

|  | **Be-exposed controls** | | **Be-hypersensitives**  **(CBD+Be-sensitized without disease)** | |
| --- | --- | --- | --- | --- |
| Allele | **N allele (90)** | **%** | **N allele (44)** | **%** |
| **0101** | 8 | 8.9% | 4 | 9.1% |
| **0103** | 1 | 1.1% | 0 | 0.0% |
| **0301** | 7 | 7.8% | 12 | 27.3%* |
| **0401** | 11 | 12.2% | 2 | 4.5% |
| **0402** | 0 | 0.0% | 1 | 2.3% |
| **0404** | 4 | 4.4% | 0 | 0.0% |
| **0405** | 1 | 1.1% | 0 | 0.0% |
| **0408** | 1 | 1.1% | 0 | 0.0% |
| **0410** | 1 | 1.1% | 0 | 0.0% |
| **0701** | 11 | 12.2% | 5 | 11.4% |
| **0801** | 0 | 0.0% | 1 | 2.3% |
| **1101** | 5 | 5.6% | 1 | 2.3% |
| **1102** | 1 | 1.1% | 1 | 2.3% |
| **1103** | 2 | 2.2% | 0 | 0.0% |
| **1104** | 1 | 1.1% | 0 | 0.0% |
| **1301** | 6 | 6.7% | 5 | 11.4% |
| **1302** | 6 | 6.7% | 2 | 4.5% |
| **1401** | 4 | 4.4% | 2 | 4.5% |
| **1406** | 1 | 1.1% | 0 | 0.0% |
| **1501** | 15 | 16.7% | 8 | 18.2% |
| **1502** | 1 | 1.1% | 0 | 0.0% |
| **1601** | 3 | 3.3% | 0 | 0.0% |

*: Uncorrected p<0.01 and Bonferroni’s corrected p>0.05 vs Be-exposed controls.

**Supplemented material table 8. Frequencies of the HLA-DRB3, -DRB4 and -DRB5 alleles in the HLA-DPGlu69-negative population.**

|  | **Be-exposed controls** | | **Be-hypersensitives**  **(CBD+Be-sensitized without disease)** | |
| --- | --- | --- | --- | --- |
| **Allele** | **N DRB3**  **Alleles (33)** | **%** | **N DRB3 alleles (22)** | **%** |
| **3* 0101** | 9 | 27.3% | 14 | 63.6%* |
| **3* 0201** | 2 | 6.1% | 0 | 0.0% |
| **3* 0202** | 16 | 48.5% | 6 | 27.3% |
| **3* 0301** | 6 | 18.2% | 2 | 9.1% |
|  | 33 | 100.0% | 22 | 100.0% |
|  |  |  |  |  |
|  | **N DRB4**  **alleles (29)** |  | **N DRB4 alleles (8)** |  |
| **4* 0101** | 6 | 20.7% | 3 | 37.5% |
| **4* 0103** | 23 | 79.3% | 5 | 62.5% |
|  | 29 | 100.0% | 8 | 100.0% |
|  |  |  |  |  |
|  | **N DRB5**  **alleles (19)** |  | **N DRB5 alleles (8)** |  |
| **5* 0101** | 16 | 84.2% | 8 | 100.0% |
| **5* 0202** | 3 | 15.8% | 0 | 0.0% |

*: Uncorrected p<0.02 and Bonferroni’s corrected p>0.05 vs Be-exposed controls
